# Prevalence of Chronic Infection by Hepatitis C Virus in Asymptomatic Population With Risk Factors in Cartagena, Colombia

**DOI:** 10.3389/fmed.2022.814622

**Published:** 2022-07-04

**Authors:** Pedro Imbeth-Acosta, Víctor Leal-Martínez, Enrique Ramos-Clason, Nehomar Pájaro-Galvis, María Cristina Martínez-Ávila, Amilkar Almanza-Hurtado, Tomás Rodríguez-Yanez, Jorge Bermudez-Montero, Oscar Vergara-Serpa, Emilio Abuabara-Franco, María Raad-Sarabia, Erika Patricia Villar-González, Steffany Isabel Tatis-Geney, Luis Adolfo Collazos-Torres, Jorge Rico-Fontalvo, Rodrigo Daza-Arnedo, Christian Pérez-Calvo, Huber Alvarado-Castell, Gabriel Hernando López Acuña

**Affiliations:** ^1^Department of Gastroenterology and Hepatology, Diagnostico y Terapeutica en Gastroenterologia y Hepatologia (DITEG), Cartagena, Colombia; ^2^Department of Internal Medicine, University of Sinu, Cartagena, Colombia; ^3^Department of Public Health and Medical Research, University of Sinu, Cartagena, Colombia; ^4^Epidemiologist, Grupo Biotoxam, Universidad de Cartagena, Cartagena, Colombia; ^5^Department of Critical Care Médicine, Gestion Salud Instituto Prestador de Salud (IPS), University of Cartagena, Cartagena, Colombia; ^6^University of Sinu, Cartagena, Colombia; ^7^Department of Family Medicine, Hospital San Vicente Fundación, Medellín, Colombia; ^8^University of Sucre, Sincelejo, Colombia; ^9^Department of Nephrology, Colombian Association of Nephrology, Medellín, Colombia; ^10^Department of Internal Medicine, Libre University, Barranquilla, Colombia; ^11^University of Cartagena, Cartagena, Colombia; ^12^Sanitas University, Bogotá, Colombia

**Keywords:** rapid diagnostic test, hepatitis C, chronic, adults, asymptomatic, prevalence

## Abstract

**Introduction:**

Infection by the hepatitis C virus (HCV) is an important cause of chronic liver disease, considered a public health problem worldwide with high morbidity and mortality due to limited access to diagnostic tests in developing countries. Only a small percentage know their infection status and receive timely treatment. It is critical to make diagnostic tests for HCV infection accessible and to provide timely treatment, which not only reduces the spread of infection but also stops the progression of HCV disease without symptoms.

**Objective:**

To determine the prevalence of chronic infection by HCV in patients with risk factors by using rapid tests in Cartagena, Colombia, and describe their epidemiological characteristics.

**Methodology:**

A cross-sectional descriptive observational study was carried out on asymptomatic adults with risk factors for HCV infection in the city of Cartagena between December 2017 and November 2019. A rapid immunochromatographic test was performed to detect antibodies, characterizing the population.

**Results:**

In total, 1,023 patients were identified who met the inclusion criteria, 58.5% women and 41.4% men, obtaining nine positive results, confirming chronic infection with viral load for HCV, finding seven cases of genotype 1b and two genotype 1a.

**Conclusion:**

In our study, a prevalence of hepatitis C infection of 0.9% was found in asymptomatic individuals with risk factors, which allows us to deduce that the active search for cases in risk groups constitutes a pillar for the identification of the disease, the initiation of antiviral therapy, and decreased morbidity and mortality.

## Introduction

Since the report of the first known case of infection with hepatitis C virus (HCV) in around the year 1989, the disease has become a public health problem worldwide with high morbidity and mortality, mainly due to the progression of chronic liver disease and hepatocellular carcinoma It occurs in about 10–20% in the next 20–30 years after diagnosis. By 2015, there were about 475,000 deaths and a global prevalence of 1.7 million cases that were added to the 71 million infected already known worldwide (1, 2).

The distribution of the HCV varies from one geographic area to another. Worldwide, 80% of all infections occur in 31 countries, contributing to more than 50% of the cases in nations, namely, China, Pakistan, Nigeria, Egypt, and India. In Colombia, epidemiological data are scarce, and the data vary according to the study population, in blood donors for the year 2000, it ranged between 0.6 and 0.97% while in hemophiliacs up to 60% have been described. For 2019, the estimated incidence was 1.0 per 100,000 inhabitants, a figure higher than previous reports, with a greater number of cases in people aged 60 years, followed by the group aged 45–59 years (3–6).

Transmission of the HCV is directly related to individuals belonging to risk groups, such as injection drug users, men who have sex with men, and people in prison (7). Its main route of infection, until 1990, was more commonly associated with direct exposure to contaminated blood through transfusions of blood components. However, with the implementation of screening for HCV in blood products, the rate of contagion has significantly decreased. In this way, the use of intravenous drugs is currently the predominant driver of new infections in the young population (8–12).

Timely diagnosis guides current treatment since the identification of the different genotypes becomes the guide for antiviral therapy. So far, eight confirmed HCV genotypes and 86 subtypes have been reported (13), with genotype 1 being the most common worldwide, responsible for 46% of all cases; the percentage and distribution of subtypes vary in each region and country (14–16).

Unfortunately, diagnosis is only achieved in about 20% of cases and of this only treatment is started in 13%. However, clinical care in the past decades has advanced considerably, and the appearance of rapid diagnostic tests that use serum, plasma, capillary whole blood, or oral fluid as matrices can be used, in place of the classical enzyme immunoassays, to facilitate the detection of antibodies against the HCV and improve access to treatment (17–21).

Antiviral therapy is curative, and the main goal of treatment is to achieve a sustained virological response, with undetectable virus RNA at 12 weeks (SVR12) or 24 weeks (SVR24) after the termination of treatment (22). In the past 5 years, HCV treatment has shifted to direct-acting antiviral regimens with superior efficacy, tolerability, and safety compared with historical interferon-based regimens. The success rate of these regimens reaches 95%, which has led to their widespread use according to the viral genotype and in populations traditionally considered difficult to treat, such as those with HIV coinfection, kidney failure, and decompensated cirrhosis (23–26). In 2016, the WHO adopted a global strategy to eliminate viral hepatitis by 2030 and obtain a 90% reduction in incident cases of hepatitis B and C, treatment of 80% of eligible people with HCV infection, and a 65% reduction in mortality. To achieve these objectives, detection, diagnosis, and sustainable access to affordable management regimes are essential (27–34).

There are few clinical studies in Colombia that reflects the prevalence of infection in large cities, such as Bogotá, Medellín, and Cali, establishing that the predominant genotype is type 1, representing 89% of cases and isolation of subtype 1b in 70% (16). Cartagena being the first tourist port in the country, the main point of arrival for cruise ships and containers at the national level, and considered the entrance bay to Latin America with tourists and cargo ships from all over the world, it is necessary to identify the prevalence of hepatitis C in the population to avoid and reduce sources of transmission and to block the progress of HCV disease without symptoms. The main objective of our study was to determine the prevalence of chronic infection by HCV in patients with risk factors using rapid capillary blood tests and to achieve a characterization of the population to allow, in the future, greater accessibility to treatment.

## Materials and Methods

This is an observational, descriptive, cross-sectional study, for the determination of the HCV in Cartagena, Colombia, conducted between December 2017 and November 2019. Asymptomatic, assumed healthy, adult patients over 18 years old with risk factors for infection with the HCV affiliated to the healthcare system who lived in Cartagena, Colombia were included (Table 1). Patients with known confirmed VHC infection were excluded, as well as people with known hepatic infections, not registered in the National Health Care affiliation system database, foreigners, or who do not reside in the city of Cartagena. The sample size was chosen from the Cartagena National Health Care Registry to select patients who met the inclusion criteria.

**Table 1:** General characteristics, diagnosis, and frequency of risk factors in patients with suspected hepatitis C virus (HCV) infection.

	***N***	**%**
**Gender**		
F	598	58.5
M	425	41.5
Age Mdn (IQR)	42 (27–54)	
18–29	287	28.1
30–39	195	19.1
40–49	194	19.0
50–59	170	16.6
60–69	112	10.9
70–79	41	4.0
80–89	24	2.3
**Education level**		
Basic	636	62.2
Medium	274	26.8
Higher	113	11.0
**Major risk factors**		
Surgeries before 1996	173	16.9
Transfusions before 1996	35	3.4
Intravenous drugs	12	1.2
Hemodialysis before 1996	9	0.9
Peritoneal dialysis	2	1.2
Transplant before 1996	1	0.1
**Minor risk factors**		
Sexual risk behaviors	922	90.1
Endoscopic studies	398	38.9
Tattoos	288	28.2
Biopsies	241	23.6
Acupuncture	53	5.2
Piercing	44	4.3
Sexual transmission disease history	17	1.7
Mother with Hepatitis C during childbirth	1	0.1
Positive diagnosis	9	0.9
1a	2	0.2
1b	7	0.7

Written consent was obtained, and the form was completed. We used the SD BIOLINE HCV (Standard Diagnostics, INC. Korea) commercial kit. This test contains a membrane precoated with HCV recombinant antigens (core, NS3, NS4, and NS5). A colloidal protein A combines with the serum sample and moves along the chromatographic membrane, forming a visible line of antigen-antibody-protein A reaction, with a high degree of specificity and sensitivity.

After cleaning with medicinal alcohol, the capillary blood sample was obtained from the pulp of one of the fingers of the non-dominant hand of the patients. A drop of blood was placed in the cavity of the kit and 4 drops of the reagent were added. The blood was expected to completely diffuse through the slot in the kit for ~20 min, and the result was interpreted according to the manufacturer's instructions: as a negative test, the presence of 1 color band within the result window, a positive test, the presence of 2 color bands (T band and C band) within the same window, an indeterminate test, the absence of color bands within the results window; in the latter case, the test was performed again.

The patients with a positive result in the rapid test underwent the confirmatory test using the real-time polymerase chain reaction (RT-PCR) method with real-time fluorescence detection for quantification of ribonucleic acid (RNA) of HCV, with a detection limit of 12 UI/ml, in addition to genotyping through amplification by PCR in real-time for the detection of the HCV genotype/subtype.

## Results

In the study period, 1,023 rapid tests were applied to detect antibodies against HCV in the at-risk population: 598 (58.5%) women and 425 (41.5%) men with an age range of 42 years [interquartile range (IQR): 27–54]. The age group with the most tests carried out was the group comprised 18–29 years with 28.1%, followed by the group 30–39 and 40–49 years with 19.1 and 19%, respectively. The educational level was more frequently basic with 62.2%, followed by medium with 26.6%.

Among the major risk factors, the most frequent were major surgeries before 1996, followed by transfusions before 1996 with 3.4%; in smaller proportions, intravenous drug use and hemodialysis were observed. Among the minor risk factors, risky sexual behavior was found more frequently with 90.1%, followed by the performance of endoscopic studies at 38.9%.

The diagnosis was positive in 0.9% of the population corresponding to nine patients. The serotype most identified was 1b in 78% of cases and 1a in 22% (Table 1). All anti-HCV-positive patients were contacted to perform HCV-RNA evaluation, to prepare them for hepatological evaluation. Out of nine positive patients, two were already treated, one was dead and six were linked to care, referred for hepatological evaluation, and then performed antiviral therapy for HCV.

When comparing the characteristics of patients with HCV infection and those healthy individuals, the frequency of women in the HCV negative group was found to be 58.8% compared with 22.2% in the positive group with a *p-*value of 0.0385; the median age in the groups was IQR 41 years (27–45) and 52 years (47–62) for the negative and positive group, respectively. The age ranges did not show a statistically significant difference, being the age group where the highest percentage of positive cases is between 40 and 69 years old with 88% of the cases. The educational level in the positive cases was basic and medium with 55.6 and 44.4%, respectively, finding no significant difference from the group of negative cases. Regarding the major risk factors, there was a greater performance of transfusions before 1996 in the positive group with 44.4 and 3.1% in the negative group with a statistically significant difference for a *p*-value of 0.0001. On the other hand, no differences were observed for the rest of the major and minor risk factors (Table 2).

**Table 2:** Comparison of characteristics of patients stratified by confirmed or unconfirmed HCV infection.

	**Negatives**	**Positives**	***p*-value**
	***N* = 1,014**	***N* = 9**	
	***n* (%)**	***n* (%)**	
**Gender**			
F	596 (58.8)	2 (22.2)	0.0385
M	418 (41.2)	7 (77.3)	
Age Mdn (IQR)	41 (27–54)	52 (47–62)	0.0342
18–29	287 (28.3)	0 (0.0)	0.2005
30–39	194 (19.1)	1 (11.1)	0.5421
40–49	191 (18.8)	3 (33.3)	0,3836
50–59	168 (16.6)	2 (22.2)	0.6265
60–69	109 (10.8)	3 (33.3)	0.0656
70–79	41 (4.0)	0 (0.0)	0.6153
80–89	24 (2.4)	0 (0.0)	0.7030
**Education level**			
Basic	631 (62.2)	5 (55.6)	0.7363
Medium	270 (26.6)	4 (44.4)	0.2594
Higher	113 (11.1)	0 (0.0)	0.6085
**Major risk factors**			
Surgeries before 1996	172 (17.0)	1 (11.1)	0.6412
Transfusions before 1996	31 (3.1)	4 (44.4)	0.0001
Intravenous drugs	12 (1.2)	0 (0.0)	0.7428
Hemodialysis before 1996	9 (0.9)	0 (0.0)	0.7766
Peritoneal dialysis	2 (0.2)	0 (0.0)	0.8339
Transplant before 1996	1 (0.1)	0 (0.0)	0.9249
**Minor risk factors**			
Sexual risk behaviors	913 (90.4)	9 (100.0)	0.3188
Endoscopic studies	396 (39.1)	2 (22.2)	0.2184
Tattoos	286 (28.2)	2 (22.2)	0.7371
Biopsies	241 (23.8)	0 (0.0)	0,1260
Acupuncture	53 (5.2)	0 (0.0)	0.4814
Piercing	44 (4.3)	0 (0.0)	0.5231
Sexual transmission disease history	17 (1.7)	0 (0.0)	0.6954
Mother with Hepatitis C during childbirth	1 (0.1)	0 (0.0)	0.9249

## Discussion

Chronic infection by HCV has become a worldwide public health problem in recent years, with significant repercussions on mortality and quality of life, as well as high healthcare costs (35–37). This is the first observational, descriptive, cross-sectional study conducted in the city of Cartagena to determine the prevalence of hepatitis C in a population at risk, and it is also the first study to describe the prevalence of HCV infection in a port city in Colombia.

In the study population, it was found that the highest prevalence of the infection was in the male gender with 77.3% compared with 41.5% in the female sex, numbers that reflect what is documented worldwide and in other Latin American countries. However, in Colombia, other studies have found a higher percentage in women (3). The foregoing reflects the variety of population characteristics affected by the virus.

Regarding the age group, the most affected population includes ages between 40 and 69 years, confirming the existing data worldwide where the highest prevalence occurs in people more than 50 years of age and, in the same way, in Colombia, where 80% of patients with chronic hepatitis C infection are in the age range of 40–69 years. Studies carried out at the national level, including the present study, agree in concluding that the fifth decade of life is the age range where we most frequently find chronic HCV infection (18). However, it should be noted that the latest data from the report of the high-cost account in Colombia shows that more and more cases appear at an early age, mainly in men, reaching an average of 42.8 years (2).

The behavior of the disease in the country has varied slightly after the implementation of resolution 1692 of 2017. If we look at the reports of the National Institute of Health in 2016, the incidence was 0.6 per 100,000 inhabitants, for a total of 182 cases per year. In 2017, this increased to 1.16 cases per 100,000 inhabitants, giving a total of 527 cases per year and for 2018, the incidence reached 1.4 cases per 100,000, representing 637 new cases (34). This rising incidence of behavior, it is believed, has been driven by the mandatory nature of notifications by SIVIGILA and a greater awareness of health personnel to identify the population at risk (5). The variation in the prevalence of hepatitis C infection in relation to the geographical area in Colombia has been demonstrated in various studies, such as the one carried out by Alvarado-Mola et al., where the prevalence in risk groups was evaluated in departments, such as Amazonas, San Andrés, Choco, and Magdalena, being 5.68, 0.66, 3.68, and 3.87%, respectively (38), and the one carried out by Martínez et al. showed in the center of the country a prevalence of 1% (39). In Cartagena, our study being the first of these investigations in the city, a prevalence of 0.9% was documented, lower values than that found in other cities in the country, but a similar figure to other countries worldwide where the prevalence oscillates about 1% (2).

Regarding the risk groups for the development of the disease, the transmission of the virus associated with healthcare, mainly during the transfusion of blood products, represented the main cause of infection until 1996, when screening techniques for donors were introduced. Although the number of infections by this route have decreased worldwide, it is still the main cause of chronic infection by HCV in our country. Beltrán et al. in his study evaluated the seroprevalence in polytransfused patients, finding a reduction of 3 times in the risk of infection between 1993 and 1995, when serological detection was introduced for HCV blood donors, achieving a reduction of more than 90% in 1995 when the coverage detection rate reached 99% (6), as did Di Filippo et al., who showed a prevalence of 83.3% in patients whose transfusion was performed before 1993, the date on which tests to detect HCV in transfusions of blood products becomes mandatory (40). In our study, the prevalence of hepatitis C associated with healthcare represents 55%, including transmission during the surgical procedure and transmission during transfusion therapy, 11 and 44%, respectively.

The use of intravenous drugs constitutes an additional risk factor for the transmission of the HCV. Worldwide, a prevalence in this population of 52.3% is reported (10); in Colombia, Toro-Tobón et al. (41) in their analysis of 667 patients with intravenous drug use in the cities of Armenia, Bogotá, Cúcuta, and Pereira, obtained 251 positives with an estimated global prevalence of HCV seropositivity of 27.3% (41), which makes us consider that in Colombia the prevalence in this population group is lower compared with other countries. In our investigation, no positive case for chronic HCV infection was detected among intravenous drug users, however, this may be due to the short history of patient exposure to this risk factor.

On the other hand, unprotected sexual behaviors constitute a risk group that has gained importance in recent years, and, although the literature shows that the percentages of transmission through this route are low. In our research, a high contagion rate was documented, coinciding with data from the 2019 patient cohort in Colombia, where the percentage increased from 11% in 2017 to 20% in 2018 (42).

In the viral genotyping part of our study population, the most frequently isolated genotype was type 1, with an identification of subtype 1b as the most common with 78% of cases, followed by 1a with 22%. These results are similar to studies carried out worldwide where genotype 1 is the most frequent with 46%, while the subtype varies in each region and country (14). In Latin America, the predominant genotype is genotype 1, with figures higher than 80% in countries, such as Mexico, Peru, and Puerto Rico, and 60% in Argentina and Brazil. Regarding the subtype, 1b was more frequent in Mexico, Venezuela, and Brazil, and 1a, in Puerto Rico and Peru (14).

In Colombia, the prevalence of genotype and subtype varies depending on the population studied. Studies, such as that of Alvarado et al., report results in blood bank donors that identify chronic HCV infection in 88% of cases due to genotype 1 (subtype 1b: 83% and subtype 1a: 5%), 9% to genotype 2 (subtype 2a: 6% and subtype 2b: 3%), and 3% to genotype 3 (38). In 2017, Santos et al. made the largest description of genotypes and subtypes of the HCV in Colombian patients, studying 1,538 patients with isolation of genotype 1 in 88.6% of cases, distributed as follows: subtype 1b, 70%; subtype 1a, 13.5%, and not determined, 5.1% of the cases; genotype 2 was found in 5.4% of the cases, genotype 3 in 2%, and genotype 4 in 4% of the cases (16).

The finding in our study of a high prevalence of genotype 1 subtype 1b is important because these have been related to the transmission of the virus associated with healthcare, such as surgical procedures or transfusion of blood products, a fact that is confirmed in our studies. Results show that the highest prevalence of HCV infection is mainly in individuals belonging to this population at risk. In contrast to the above, the latest report from the high-cost account for the 2019 cohort reports a high prevalence of genotype 4, ranking second with 19% of cases (43). In our study, we did not find any case due to the genotype in question, a fact that can be explained by the association of this genotype with the human immunodeficiency virus (HIV), as demonstrated by Toro-Tobón and Berbesi-Fernández in their most recent study (44). In our investigation, patients with HIV infection were not screened. Other risk factors, such as tattoos or body piercing, were frequent in our study population, however, no positive cases were found in this risk group.

## Data Availability Statement

The original contributions presented in the study are included in the article/supplementary material, further inquiries can be directed to the corresponding author/s.

## Ethics Statement

The studies involving human participants were reviewed and approved by Universidad del Sinu. The patients/participants provided their written informed consent to participate in this study.

## Author Contributions

JB-M, OV-S, EA-F, MR-S, EV-G, ST-G, and LC-T collected the data. PI-A, VL-M, NP-G, AA-H, and TR-Y prepared the manuscript. ER-C and MM-Á performed the statistical analysis and interpreted the data. JR-F, RD-A, CP-C, HA-C, and GL interpreted the data and critically revised the manuscript. All authors contributed to the article and approved the submitted version.

## Conflict of Interest

The authors declare that the research was conducted in the absence of any commercial or financial relationships that could be construed as a potential conflict of interest.

## Publisher's Note

All claims expressed in this article are solely those of the authors and do not necessarily represent those of their affiliated organizations, or those of the publisher, the editors and the reviewers. Any product that may be evaluated in this article, or claim that may be made by its manufacturer, is not guaranteed or endorsed by the publisher.
